# Residual Stress and Warping Analysis of the Nano-Silver Pressureless Sintering Process in SiC Power Device Packaging

**DOI:** 10.3390/mi15091087

**Published:** 2024-08-28

**Authors:** Wenchao Tian, Dexin Li, Haojie Dang, Shiqian Liang, Yizheng Zhang, Xiaojun Zhang, Si Chen, Xiaochuan Yu

**Affiliations:** 1 School of Electro-Mechanical Engineering, Xidian University, Xi’an 710071, China; 22041212901@stu.xidian.edu.cn (D.L.); d2100710345@outlook.com (H.D.); 22041212746@stu.xidian.edu.cn (S.L.); 2 State Key Laboratory of Electromechanical Integrated Manufacturing of High-Performance Electronic Equipments, Xi’an 710071, China; 3 The Thirteenth Research Institute of China Electronics Group Corporation, Shijiazhuang 050000, China; zhangyz@cetc13.cn (Y.Z.); zhangxjcetc13@cetc13.cn (X.Z.); 4 The Fifth Electronics Research Institute of Ministry of Industry and Information Technology, Guangzhou 510000, China; chensiceprei@yeah.net; 5 Shandong Junyu Electronic Technology Co., Ltd., Linyi 276100, China; hyang.yu@cnjunyong.com

**Keywords:** nano-silver paste, pressureless sintering, SiC, residual stress, warpage

## Abstract

Chip bonding, an essential process in power semiconductor device packaging, commonly includes welding and nano-silver sintering. Currently, most of the research on chip bonding technology focuses on the thermal stress analysis of tin–lead solder and nano-silver pressure-assisted sintering, whereas research on the thermal stress analysis of the nano-silver pressureless sintering process is more limited. In this study, the pressureless sintering process of nano-silver was studied using finite element software, with nano-silver as an interconnect material. Using the control variable method, we analyzed the influences of sintering temperature, cooling rate, solder paste thickness, and solder paste area on the residual stress and warping deformation of power devices. In addition, orthogonal experiments were designed to optimize the parameters and determine the optimal combination of the process parameters. The results showed that the maximum residual stress of the module appeared on the connection surface between the power chip and the nano-silver solder paste layer. The module warping deformation was convex warping. The residual stress of the solder layer increased with the increase in sintering temperature and cooling rate. It decreased with the increase in coating thickness. With the increase in the coating area, it showed a wave change. Each parameter influenced the stress of the solder layer in this descending order: sintering temperature, cooling rate, solder paste area, and solder paste thickness. The residual stress of the nano-silver layer was 24.83 MPa under the optimal combination of the process parameters and was reduced by 29.38% compared with the original value of 35.162 MPa.

## 1. Introduction

Since the beginning of the 21st century, with the rapid development of semiconductors and integrated circuits, power electronic devices have been improved in terms of miniaturization, high performance, high operating temperature, and high power density [1,2,3,4]. The third generation of high-power semiconductor chips represented by silicon carbide (SiC) and gallium nitride (GaN) emerge with power devices with high bandgap width, electric field breakdown strength, and electrical and thermal conductivity [5,6]. As the mainstream device of modern power electronics technology, the insulated gate bipolar transistor (IGBT) is widely used due to its excellent electrical performance and low expenditure required for activation. In systems with low fault tolerance, such as aircrafts, high-speed trains, and hybrid electric vehicles, any sudden failure of the power devices can be fatal.

Warping and residual stress, the main causes of reliability problems, exist not only in the normal operation of devices but also in their fabrication and packaging process, which can lead to the so-called yield problem [7,8]. In the packaging process of power devices, warping and residual stress are inevitable due to the mismatch between the coefficients of thermal expansion (CTE) of the package materials [9]. The residual stress inside the module will significantly impact its reliability and operating life. Module warping not only causes direct bonded copper (DBC) substrate delamination cracking, solder layer cracks, and other fatigue failures but also reduces the contact area between the connecting layers, thus increasing the thermal resistance of the system and reducing the heat dissipation performance. The connections between the chip and the DBC substrate and between the DBC substrate and the heat dissipation substrate are two main elements that generate warping and residual stress during the packaging of power devices. Chip bonding, an essential process in power semiconductor packaging, commonly includes welding and nano-silver sintering. Traditional electronic packaging connection materials such as solder and conductive adhesive cannot be used in high-temperature applications because their operating temperature is less than 175 °C, and degradation phenomena such as creep easily occur under large temperature changes [10]. Nano-silver sintering has been widely used because of its excellent reliability and adaptability to work at high temperatures.

Xu et al. [11] studied the effect of component thickness on the residual stress and warpage of IGBT module chips in the reflow soldering process. Addagarla et al. [12] established the flip-chip model. Using the transient thermal structure coupling field analysis method, they studied the stress and deformation distribution in the chip–substrate packaging process. Kang et al. [13] analyzed the effects of solder spacing, package size, and substrate thickness on warping. The results showed that the appropriate combination of solder spacing, package size, and substrate thickness can reduce the warping of the package structure. Zhou et al. [7] studied the effect of copper layer patterns on warping and residual stress in the IGBT reflow soldering process. The results showed that the maximum equivalent stress of the patterned and unpatterned copper layer appeared in the ceramic layer of the DBC substrate after reflow soldering, and the copper layer patterns of the DBC substrate had little effect on the residual stress and warping of the IGBT module. Liu et al. [14] studied the stress distribution on the chip of a double-sided silver sintering power module under different sintering pressures. Bai et al. [15] found that a nano-silver solder paste could be sintered at a temperature as low as 190 °C without pressure, thus achieving the goals of low-temperature pressureless sintering and high-temperature service of the solder. At present, most of the research on the bonding process of power device chips focuses on the thermal stress analysis of tin–lead solder and nano-silver press-assisted sintering. There are few studies on the thermal stress analysis of the nano-silver pressureless sintering process, and the existing studies considered a single factor or no more than three factors to analyze the influence of the process parameters on the sintering joint strength.

For this reason, the pressureless sintering process of a nano-silver paste was studied by using a nano-silver paste as the bonding medium. The effects of sintering temperature, cooling rate, solder paste thickness, and solder paste area on the residual stress and warping deformation of power devices were studied by using ANSYS 19.2 finite element analysis software. At the same time, an orthogonal experiment was designed to carry out an orthogonal optimization simulation [16] to analyze and classify the influence of each parameter on the stress of the solder layer. The optimal combination of the process parameters was clearly defined to provide guidance for setting the process parameters of pressureless nano-silver sintering in future practical projects.

## 2. Model and Parameters

In this study, ANSYS software was used for the three-dimensional finite element simulation of the power module, which consists of eight IGBT chips, chip solder layers, a DBC substrate, a gold germanium (AuGe) layer, and a cooling bottom plate. The cooling substrate is connected to the copper layer under the DBC substrate through the AuGe layer. The bottom of the power chip is connected to the upper copper layer of the DBC substrate by the under-chip solder layer [17].

**Figure 1:**
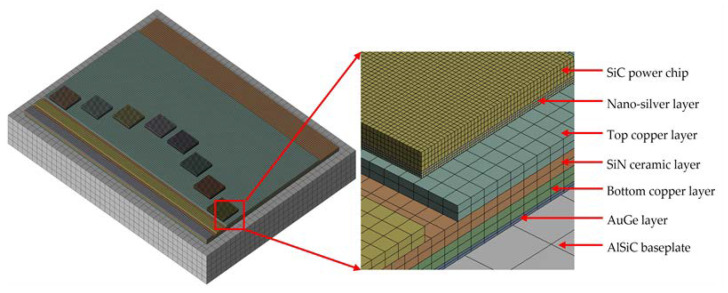
Finite element mesh model of the power module.

The 3D model of the power module and the combination of its different layers are shown in Figure 1, and Table 1 lists the geometric parameters of each part of the module. The model structure analyzed in this study was relatively simple, with no significant curvature boundary. A mapping grid was used in the meshing method. Because the ratio of the length–width to the thickness of each layer structure was too large, each layer structure was divided into at least two layers in the thickness direction.

The thermodynamic parameters of various materials in the model at room temperature are shown in Table 2, including density, Young’s modulus, Poisson’s ratio, coefficient of thermal expansion, thermal conductivity, and specific heat capacity. Because nano-silver particles have an enormous specific surface area and a minimal surface curvature radius, they can begin to melt at 100 °C and produce inelastic deformation. Young’s modulus will change with the temperature. Table 3 shows the specific values of Young’s modulus of nano-silver varying with the temperature [14,18]. The finite element simulation considered the nonlinear mechanical behavior, and the Anand viscoplastic constitutive model was used to describe the mechanical properties of nano-silver solder [11]. Anand first proposed the Anand constitutive model and then developed it [19]. This study refers to the Anand model of sintered silver proposed by Chen et al., and the parameters are shown in Table 4 [20]. The room temperature of 25 °C was set as the reference temperature, at which the nano-silver solder was in the state of zero stress and strain. Because the CTE and Young’s modulus of the bottom plate have a great influence on the accuracy of the finite element model, this study refers to the values of CTE and Young’s modulus of the AlSiC bottom plate at different temperatures obtained by Gao et al., and the parameters are shown in Table 5 [21].

**Figure 2:**
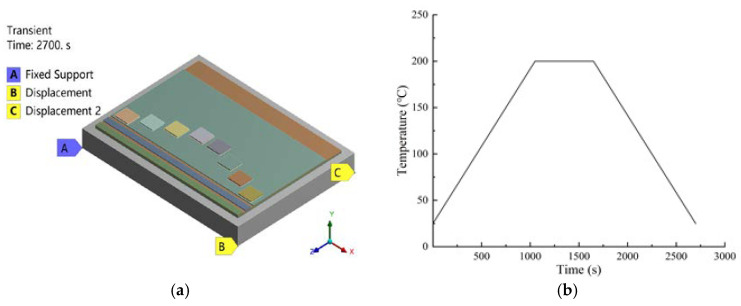
Boundary conditions. (**a**) Three-point constraint approach; (**b**) Temperature load.

In the process of finite element simulation, to avoid excessive thermal expansion constraints, the three-point constraint method was adopted for the base plate to limit the six degrees of freedom of the model. As shown in Figure 2a, point A limited the displacement in the X, Y, and Z directions, point B restricted the displacement in the Y and Z directions, and point C restricted the displacement in the Y direction. The temperature load shown in Figure 2b was applied to the model.

## 3. Effect of the Chip Sintering Process on Warpage and Residual Stress in the Power Modules

### 3.1. Warpage and Residual Stress Analysis in the Power Modules after Sintering

The connection between the chip and the DBC substrate was influenced by the different thermal expansion coefficients of the SiC chip, the nano-silver solder paste, and the DBC substrate’s copper layer. The chip connection layer as subjected to a shear force along the connection surface in the cooling process. When the whole structure was warped, more complex forces and stress concentration in the corners affected the connection layer. To accurately describe the stress state of the solder layer, the literature [24,25] proposed that the classical von Mises model can explain the inelastic behavior of many materials. The main reason for the failure of the chip connection was the excessive plastic deformation of the solder layer in the sintering process; so, the stress state of the solder layer could be obtained from the equivalent stress results.

**Figure 3:**
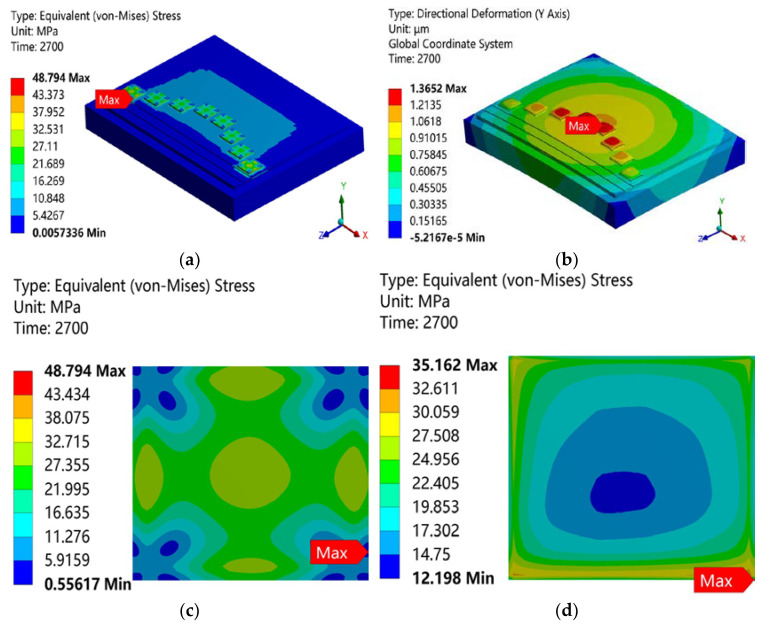
Equivalent stress and deformation cloud diagram of the power module after sintering: (**a**) overall equivalent stress distribution diagram, (**b**) overall deformation distribution diagram, (**c**) chip equivalent stress distribution diagram, (**d**) solder equivalent stress distribution diagram.

During this finite element simulation, all components of the module were initially flat, and the initial residual stresses of the DBC substrate, silver solder paste, and bottom plate were ignored. The finite element simulation results are shown in Figure 3. It can be seen from the overall stress cloud diagram of the module in Figure 3a that the maximum residual stress was 48.794 MPa and appeared in the power chip. Because the thermal mismatch between the power chip and the nano-silver layer was the most serious in the power module structure, the maximum residual stress of the module occurred on the connection surface between the power chip and the nano-silver layer. From the warping cloud diagram in Figure 3b, it can be seen that the closer to the center, the more serious the warping was. The whole module showed convex warping, and the maximum warping value was 1.3652 μm. It can be seen from the stress cloud diagram of the chip in Figure 3c that the stress distribution of the chip was large in the center and small in the corners around it. The maximum stress occurred on the contact surface between the chip and the solder, and the maximum residual stress was 48.794 MPa. Because the thermal expansion coefficient of the solder paste was greater than that of the chip, the solder paste shrank faster. It can be seen from the stress cloud diagram of the nano-silver layer in Figure 3d that the residual stress of the solder layer diffused from the center of the solder layer to its edges in a ring, and the maximum stress of the solder layer was 35.162 MPa, which occurred in the corners of the solder layer. This means that the solder was prone to stress failure in the corner areas during the working process. The trend of the residual stress distribution of solder and chip was consistent with previous numerical analysis results [21]. The stress and warping distribution results for the power devices during the press-assisted sintering process are analyzed in detail in Appendix A.

### 3.2. Effect of the Process Parameters on Warping and Residual Stress

The bonding of chips on power device can present welding difficulties, being prone to cavity formation and affected by excessive bonding layer thickness, warping, and other problems. Selecting better process parameters could be conducive to reducing residual stress and warping.

This study mainly focused on the four parameters of sintering temperature, cooling rate, and nano-silver coating thickness and area. Using the control variable method, only one process parameter was changed, and the effects of the different process parameters on sintering warping and residual stress were studied and analyzed through horizontal comparison. The simulation test design is shown in Table 6.

**Figure 4:**
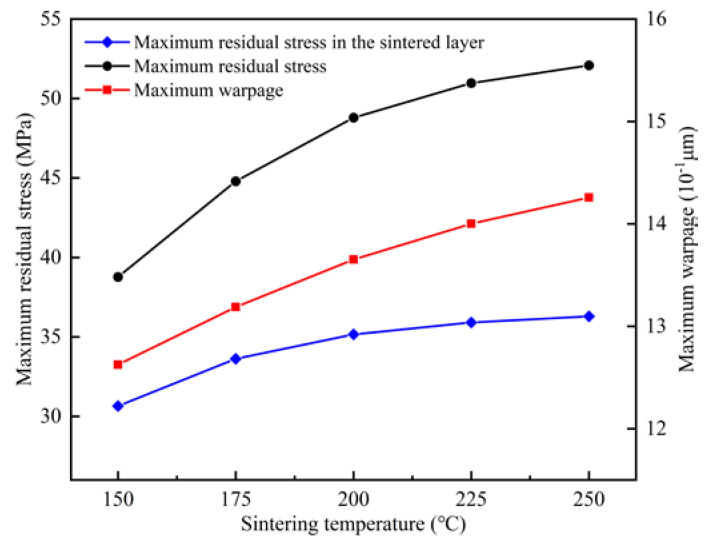
Effect of sintering temperature on module warpage and residual stress.

**Figure 5:**
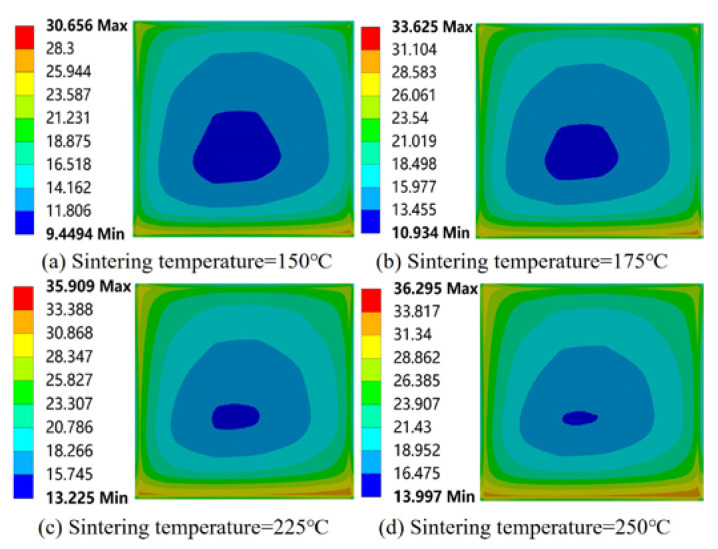
Effect of sintering temperature on residual stresses in solder paste layer.

It can be seen from Figure 4 that with the increase in sintering temperature, the maximum residual stress of the module gradually increased from 38.773 MPa to 52.089 MPa, and the maximum warping gradually increased from 1.2625 μm to 1.4258 μm. Both the maximum residual stress and warping gradually increased with the increase in sintering temperature, but the increase was gradually reduced. This is because with the increase in temperature, the stiffness of the bottom plate and the resistance to deformation decreased. This is consistent with the variation in substrate warping with the increase in sintering temperature observed by Guo et al. using the finite element model [26]. Combining Figure 4 and Figure 5, it was found that the stress distribution in the center of the solder paste layer developed towards the direction of high stress with the increase in sintering temperature, and the area of low stress in the center became progressively smaller. This change might reduce the ability of the solder paste connection layer to withstand loads such as those caused by thermal cycling or thermal shock during the working process of the module and reduce the life of the module. The maximum residual stress of the solder layer increased with the increase in sintering temperature. Li et al. [27] experimentally studied the average shear strength of a nano-silver paste at different sintering temperatures and showed that at a lower sintering temperature a higher average shear strength could be obtained under the condition of keeping other process parameters unchanged. The effect of the sintering temperature on the solder fillet shape is detailed in Appendix B.

**Figure 6:**
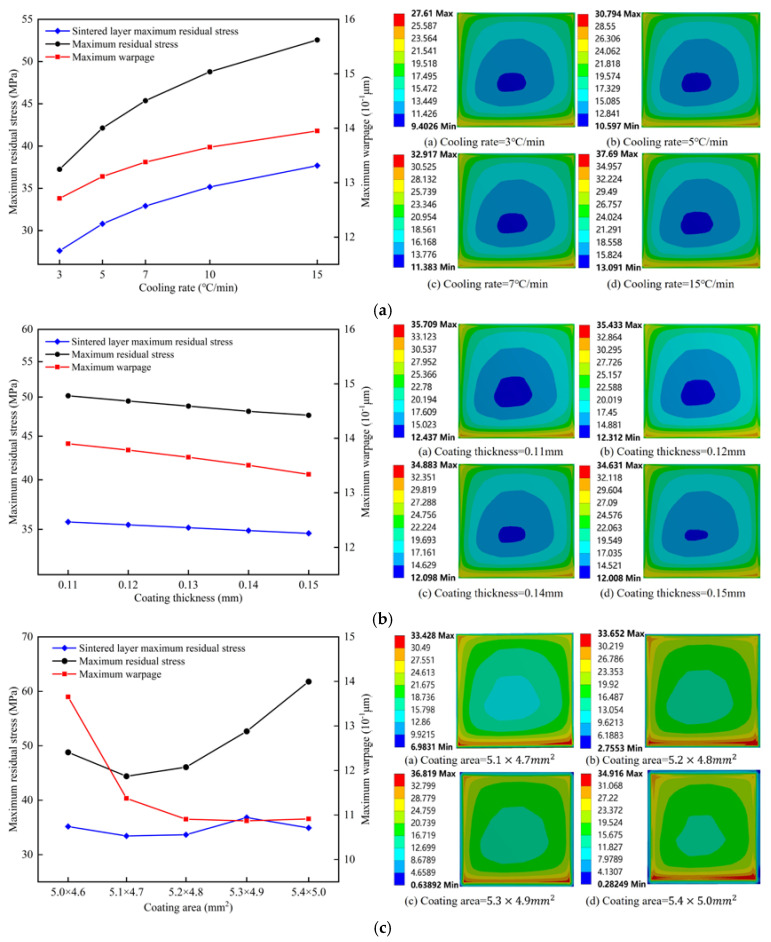
Effect of other process parameters on sintering warping and residual stress. (**a**) Cooling rate; (**b**) Coating thickness; (**c**) Coating area.

Figure 6 shows the influence of other process parameters (cooling rate, coating thickness, and coating area) on sintering warping and residual stress. Observing the warping of the module, it can be seen that the warping of the module increased with the increase in the cooling rate, but warping caused by the sintering process of the module chip could be reduced by increasing the area and thickness of the solder paste layer.

It can be seen from Figure 6a that the residual stress of the chip and solder paste layer increased with the increase in cooling rate. Changes in the cooling rate had little effect on the stress distribution in the center of the solder layer, and large stress changes were mainly concentrated in the corners of the solder layer. These changes in cooling rate may have no obvious effect on module manufacturing but may reduce the ability of the solder layer to bear loads such as during thermal shock and reduce the life of the module while working. However, if the cooling rate is too slow in practical engineering, the grain size of the solder paste will increase, and gold intergeneric compounds will grow too much, decreasing the solder layer’s fatigue resistance. Therefore, it is necessary to adjust the cooling rate in practical engineering to reduce the solder layer’s manufacturing defects and improve its reliability.

It can be seen from Figure 6b that the residual stress of the chip and the solder paste layer decreased continuously with the increase in the thickness of the solder paste. This trend is consistent with the observed trend of residual stress of the chip and solder paste layer with the increase in solder paste thickness [7]. If the interconnection interface is too thick in chip manufacturing, the chip manufacturing cost will significantly increase, and the current requirements for the miniaturization of electronic products will not be met. On the contrary, if the interconnection interface is too thin, low connection reliability will occur. In fact, when the chip is mounted on the substrate, if the thickness of the solder paste is not sufficient due to the extrusion of the chip, the solder paste will be unevenly dispersed between the chip and the substrate, and the sintering quality will be sharply reduced.

It can be seen from Figure 6c that with the increase in the coating area, the residual stress of the chip layer first decreased and then increased, and the residual stress of the solder paste layer changed in a wavy way. The residual stress of the solder paste layer did not show a single trend with the change in the coating area. For the solder paste coating area, we could not simply conclude that the more, the better, or the less, the better, but that an appropriate coating area existed. Qi et al. [28] experimentally studied the influence of the nano-silver interconnect area on bond strength. The results showed that with the increase in the interconnect area, the bond strength of the joint showed a wavy decreasing trend. Considering that excessive area and thickness of the solder paste layer would increase the manufacturing cost and the difficulty of the welding process and easily produce voids and cracks, it is essential to choose appropriate area and thickness of the solder paste layer.

### 3.3. Orthogonal Analysis of the Sintering Process Parameters

According to the analysis in Section 3.2, optimal ranges for the process parameters of the nano-silver sintering process were obtained. However, these process parameters interact with each other. Therefore, a multi-factor orthogonal experiment was designed to analyze the influence of sintering temperature, cooling rate, nano-silver solder paste thickness, and nano-silver solder paste area on the sintering residual stress of the solder bonding layer. The results will provide a basis for the optimal design of the IGBT module chip bonding process.

The thickness of the nano-silver solder paste, its area, the sintering temperature, and the cooling rate were used as test factors, and three levels (levels 1, 2, 3) were selected for each test factor based on the minimum residual stress. The experiment was designed according to the orthogonal table of four factors and three levels, and the horizontal distribution of each factor is shown in Table 7. The thickness of the nano-silver solder paste is indicated as A, the area of the nano-silver solder paste as B, the sintering temperature is expressed as C, and the cooling rate as D.

According to the simulation results, the maximum residual stress of the solder layer was obtained when the temperature was reduced to 25 °C; the range analysis method was used to analyze the residual stress. The results are shown in Table 8.

Range analysis is a common method for the analysis of orthogonal test results. The magnitude of the ranges reflects the influence of the investigation factors, and an extensive range means that changes in that investigation factor have a significant influence on the test results, identifying the main factor affecting the experimental results. On the contrary, a small range identifies a secondary factor. First of all, the maximum residual stress of the solder layer in repeated tests at the corresponding level of each factor was expressed as *K_i_* (*i* represents different levels of each factor). In addition, the average residual stress of the solder layer was called the index average, which was expressed as *k_i_*. The best level of each factor could be obtained with *k_i_* and *K_i_*, and the relationship between them is shown in Equation (1).

(1) $$k_{i}=K_{i}/3$$

Secondly, to compare the test indexes of the various factors at different levels, the range *R_i_* was defined to describe the significance of each factor in the orthogonal test. The range *R_i_* is the difference between the maximum and the minimum *K_i_* for each factor, as shown in Equation (2).

(2) $$R_{i}=k_{i\max}-k_{i\min}$$

According to the range analysis results in Table 8, it can be concluded that the optimal horizontal combination was A_2_B_2_C_3_D_3_, and the residual stress value of the sintered nano-silver solder layer under the optimal parameter combination was 24.83 MPa. Compared with the original value of 35.162 MPa, the stress was reduced by 29.38%. According to the range results, it can be seen that based on their effects on chip silver sintering, the process parameters can be classified in the following order with decreasing priority: C (sintering temperature), D (cooling rate), B (coating area), and A (coating thickness).

## 4. Conclusions

In this study, a 3D finite element model of the power module was successfully established, and the influence of the nano-silver pressureless sintering process parameters on the residual stress and warpage of the module was studied. The control variable method and orthogonal test analysis were used to reduce the number of tests scientifically and rationally, avoid unsignificant tests, and minimize the residual stress and warping. The main findings are as follows:

1. After chip reflow welding, the power module as a whole showed convex warping. The maximum residual stress of the module appeared on the connection surface between the power chip and the solder paste layer. The stress distribution in the nano-silver solder paste layer increased from the center to the edges of the distribution circle, and the maximum residual stress appeared in the corners of the solder paste.
2. The overall residual stress increased with the increase in sintering temperature and cooling rate. It decreased with the increase in coating thickness. It first decreased and then increased with the increase in coating area. Increasing the area and thickness of the solder paste layer could reduce the warping caused by the module in the chip welding process.
3. The residual stress of the solder layer increased with the increase in sintering temperature and cooling rate. It decreased with the increase in coating thickness. With the increase in the coating area, it showed a wavy change.
4. The orthogonal test analysis showed that the chip silver sintering process parameters can be classified in the following order with decreasing priority: sintering temperature, cooling rate, coating area, and coating thickness. The optimized chip welding parameters were as follows: the thickness of the solder paste was 0.14 mm, the area of the solder paste was 5.1 × 4.7 mm^2^, the sintering temperature was 150 °C, and the cooling rate was 5 °C/min. Under this optimal combination of the process parameters, the residual stress of the solder layer obtained by sintering was 24.83 MPa and was reduced by 29.38% compared with the original value of 35.162 MPa. This study provides a reference for selecting the process parameters of power module nano-silver sintering without pressure.

This study investigated the influence of various process parameters on warping and residual stress distribution in the chip connection layer during the sintering process of power device chips. However, this research assumed that the initial state of the DBC substrate was characterized by zero stress and strain and did not take into account the porosity of nano-silver. As a result, it did not fully capture the variability of the actual processing conditions. Future studies will explore the effects of the substrate’s initial state and the porosity of nano-silver on the properties of the connected layer according to the actual process conditions.

## Figures and Tables

**Table 1:** Geometrical size of each layer.

Layer	Materials	Length (mm)	Width (mm)	Thickness (mm)
Colling baseplate	AlSiC	60.0	45.0	6.0
Baseplate solder layer	AuGe	55.0	40.0	0.1
Down copper	Cu	55.0	40.0	0.3
Ceramic layer	Si_3_N_4_	55.0	40.0	0.32
Up copper	Cu	55.0	28.0	0.3
Chip solder layer	Nano-silver	5.0	4.6	0.13
IGBT chip	SiC	5.0	4.6	0.36

**Table 2:** Material parameters of each component.

Materials	Density ρ (kg/m^3^)	Young’s Modulus E (GPa)	Poisson’s Ratio ν	CTE α (1 × 10^−6^/K)	Thermal Conductivity λ (W/m·K)	Specific Heat Capacity c (J/(kg·°C))
SiC [22]	3200	410	0.14	4.5	370	800
Nano-silver [18]	10500	Table 3	0.37	19.6	240	234
Cu [11]	8950	110	0.34	16.4	385	385
Si_3_N_4_ [17]	3200	320	0.25	3	80	710
AuGe [23]	14670	68	0.32	13.4	44.4	130
AlSiC [21]	2960	Table 5	0.4	Table 5	200	750

**Table 3:** Young’s modulus of low-temperature sintered nano-silver.

Temperature (°C)	25	60	120	150	250
Young’s modulus (GPa)	6.28	4.52	2.64	1.58	0.5

**Table 4:** Anand model material parameters of nano-silver.

Parameters	Value	Definition
S_0_ (MPa)	2.93	Initial value of deformation resistance
Q/R (K)	5706.3	Activation energy/Boltzmann constant
A (1/s)	9.81	Pre-exponential factor
ξ	12	Stress multiplier
m	0.6572	Strain rate sensitivity of stress
h_0_ (MPa)	14600	Hardening coefficient
ŝ (MPa)	101.7	Coefficient for deformation resistance saturation value
n	0.00326	Strain rate sensitivity of saturation value
α	1	Strain rate sensitivity of hardening coefficient

**Table 5:** Structural properties of AlSiC.

Temperature (°C)	Young’s Modulus (GPa)	CTE(1E-6/K)
50	192.68	3.65
100	189.74	5.67
150	183.26	6.59
200	178.70	7.16
250	176.19	7.48
300	167.81	7.62

**Table 6:** Simulation of the experimental design.

Serial Number	Sintering Temperature (°C)	Heating Rate (°C/min)	Nano-Silver Thickness (mm)	Nano-Silver Area (mm^2^)
1	150	10	0.13	5.0 × 4.6
2	175	10	0.13	5.0 × 4.6
3	200	10	0.13	5.0 × 4.6
4	225	10	0.13	5.0 × 4.6
5	250	10	0.13	5.0 × 4.6
6	200	10	0.13	5.0 × 4.6
7	200	10	0.13	5.0 × 4.6
8	200	10	0.13	5.0 × 4.6
9	200	10	0.13	5.0 × 4.6
10	200	10	0.13	5.0 × 4.6
11	200	3	0.13	5.0 × 4.6
12	200	5	0.13	5.0 × 4.6
13	200	7	0.13	5.0 × 4.6
14	200	10	0.13	5.0 × 4.6
15	200	15	0.13	5.0 × 4.6
16	200	10	0.11	5.0 × 4.6
17	200	10	0.12	5.0 × 4.6
18	200	10	0.13	5.0 × 4.6
19	200	10	0.14	5.0 × 4.6
20	200	10	0.15	5.0 × 4.6
21	200	10	0.13	5.0 × 4.6
22	200	10	0.13	5.1 × 4.7
23	200	10	0.13	5.2 × 4.8
24	200	10	0.13	5.3 × 4.9
25	200	10	0.13	5.4 × 5.0

**Table 7:** Orthogonal experimental design, factors and levels.

Factor Level	A (mm)	B (mm^2^)	C (°C)	D (°C/min)
1	0.15	5.2 × 4.8	200	10
2	0.14	5.1 × 4.7	175	7
3	0.13	5.0 × 4.6	150	5

**Table 8:** Range analysis table of orthogonal test results.

Test Number	Factor				Maximum Residual Stress (MPa)
	A	B	C	D	
1	0.15	5.2 × 4.8	200	10	32.723
2	0.15	5.1 × 4.7	175	7	28.616
3	0.15	5.0 × 4.6	150	5	27.172
4	0.14	5.2 × 4.8	175	5	27.691
5	0.14	5.1 × 4.7	150	10	27.783
6	0.14	5.0 × 4.6	200	7	32.668
7	0.13	5.2 × 4.8	150	7	27.116
8	0.13	5.1 × 4.7	200	5	29.116
9	0.13	5.0 × 4.6	175	10	33.625
*K*_1_	88.511	87.530	94.507	94.131	
*K*_2_	88.142	85.515	89.932	88.400	
*K*_3_	89.857	93.465	82.071	83.979	
*k*_1_	29.504	29.177	31.502	31.377	
*k*_2_	29.381	28.505	29.977	29.467	
*k*_3_	29.952	31.155	27.357	27.993	
*R_i_*	0.572	2.650	4.145	3.384	
Sequence	C > D > B > A				
Optimal combination	A_2_B_2_C_3_D_3_				24.83

**Figure A1:**
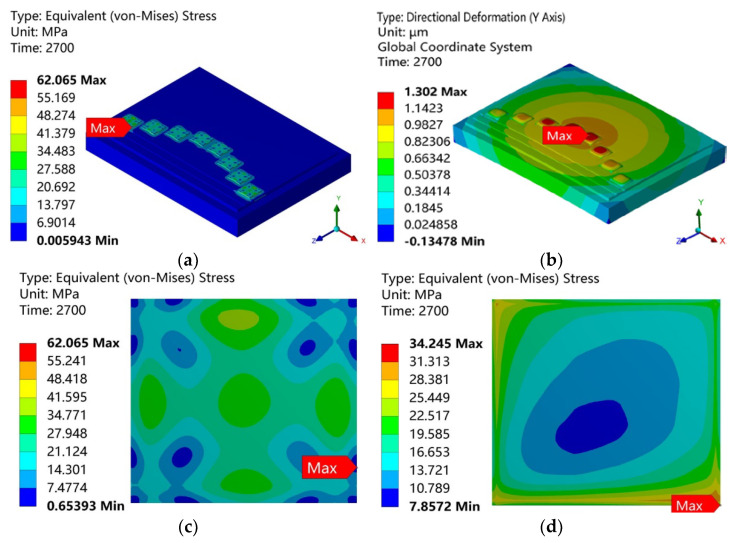
Equivalent stress and deformation cloud diagram of the power module under the condition of applying 4 MPa sintering pressure. (**a**) Overall equivalent stress distribution diagram, (**b**) overall deformation distribution diagram, (**c**) chip equivalent stress distribution diagram, (**d**) solder equivalent stress distribution diagram.

**Figure A2:**
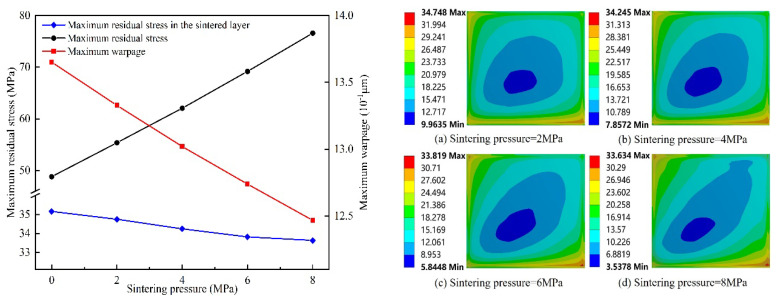
Effect of sintering pressure on module warpage and residual stress.

**Figure A3:**
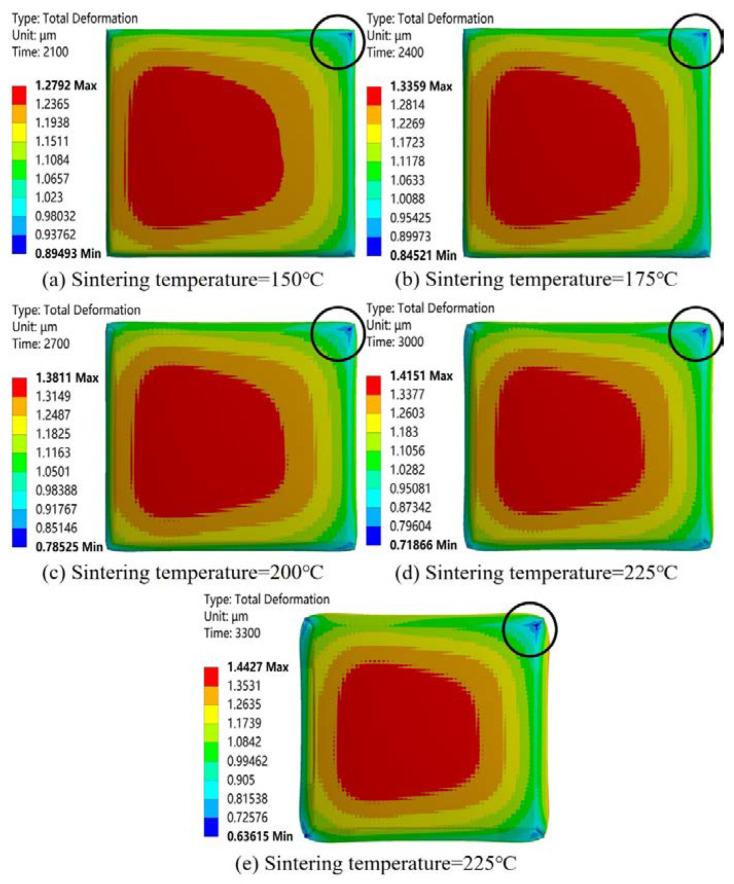
Variation of solder fillet shape with sintering temperature.

## Data Availability

Data are contained within the article.

## Appendix A. Pressure Sintering of Nano-Silver

The finite element simulation results of the power module under pressure sintering of nano-silver are shown in Figure A1. From the overall stress cloud diagram of the module in Figure A1a, it can be seen that the residual stress distribution range of the copper layer on the DBC substrate under pressure sintering was smaller than that under pressureless sintering. The maximum residual stress of the whole module was 62.065 MPa, and the maximum residual stress appeared on the connection surface between the power chip and the nano-silver solder layer. From the warping cloud diagram in Figure A1b, it can be seen that the closer to the center, the more serious the warping is. The whole module showed convex warping, and the maximum warping value was 1.302 μm. From the stress cloud diagram of the chip in Figure A1c, it can be seen that the stress of the chip was large in the center and small in the corners. Maximum stress was positioned on the contact surface between the chip and the solder, and the maximum residual stress was 62.065 MPa. From the stress cloud diagram of the nano-silver layer in Figure A1d, it can be seen that the residual stress of the solder layer diffused from the center to the edges of the solder in an elliptical shape, and the maximum stress of the solder layer was 35.162 MPa, which occurred in the corners of the solder layer. In general, regardless of whether pressureless sintering (Figure 3) or pressure sintering (Figure A1) was used, the maximum residual stress and warping positions in the power module as a whole and in each component were consistent.

It can be seen from Figure A2 that both the maximum residual stress and the warping of the module changed linearly with the increase in sintering pressure. The maximum residual stress gradually increased from 48.794 MPa to 76.603 MPa, and the maximum warping gradually decreased from 1.3652 μm to 1.2468 μm. The maximum residual stress of the solder layer decreased with the increase in sintering pressure. Lu et al. [29] experimentally investigated the effect of sintering pressure on the strength of nano-silver joints. The results showed that the bond strength of the joint increased with the increase in sintering pressure. The above analysis showed that applying pressure in the process of chip sintering can effectively reduce the residual stress of the solder layer and the warping of the module but will greatly increase the residual stress of the chip, which will greatly affect the reliability and life of the power chip when the module is working.

## Appendix B. Variation of the Solder Fillet Shape

By observing the deformation of the solder layer at different sintering temperatures, it was seen that with the increase in sintering temperature, the shape of the solder layer corners gradually changed from sharp to rounded. This is because the surface energy of nano-silver tended to be minimized as the sintering temperature increased during the sintering process. The shape with sharp corners can be easily transformed into one with rounded corners with lower energy due to its higher surface energy.

## References

[1] Tian W., Wu S., Li W. (2023). Research of Vertical via Based on Silicon, Ceramic and Glass. Micromachines.

[2] Tian W., Li P., Yuan L. (2018). Research and analysis of MEMS switches in different frequency bands. Micromachines.

[3] Rafin S., Ahmed R., Haque M., Hossain M., Haque M., Mohammed O. (2023). Power Electronics Revolutionized: A Comprehensive Analysis of Emerging Wide and Ultrawide Bandgap Devices. Micromachines.

[4] Sheng K., Williams B.W., Finney S.J. (2000). A review of IGBT models. IEEE Trans. Power Electron..

[5] Yan L., Liu P., Xu P., Tan L., Zhang Z. (2023). Reliability Analysis of Flip-Chip Packaging GaN Chip with Nano-Silver Solder BUMP. Micromachines.

[6] Qian J., Shi L., Jin M., Bhattacharya M., Shimbori A., Yu H., Houshmand S., White M.H., Agarwal A.K. (2024). An Investigation of Body Diode Reliability in Commercial 1.2 kV SiC Power MOSFETs with Planar and Trench Structures. Micromachines.

[7] Zhou Y., Xu L., Liu S. (2015). Optimization for warpage and residual stress due to reflow process in IGBT modules based on pre-warped substrate. Microelectron. Eng..

[8] Liu Y. (2012). Power Electronic Packaging: Design, Assembly Process, Reliability and Modeling.

[9] Deng S.-S., Hwang S.-J., Lee H.-H. (2016). Temperature prediction for system in package assembly during the reflow soldering process. Int. J. Heat Mass Transf..

[10] Siow K.S. (2014). Are sintered silver joints ready for use as interconnect material in microelectronic packaging?. J. Electron. Mater..

[11] Xu L., Liu Y., Liu S. (2014). Modeling and simulation of power electronic modules with microchannel coolers for thermo-mechanical performance. Microelectron. Reliab..

[12] Addagarla A., Prasad N.S. (2012). Finite element analysis of flip–chip on board (FCOB) assembly during reflow soldering process. Solder. Surf. Mt. Technol..

[13] Kang J.S. (2020). Parametric study of warpage in PBGA packages. Int. J. Adv. Manuf. Technol..

[14] Liu Y., Zhang H., Wang L., Fan X., Zhang G., Sun F. (2019). Stress analysis of pressure-assisted sintering for the double-side assembly of power module. Solder. Surf. Mt. Technol..

[15] Bai J.G., Zhang Z.Z., Calata J.N., Lu G.Q. (2006). Low-Temperature Sintered Nanoscale Silver as a Novel Semiconductor Device-Metallized Substrate Interconnect Material. IEEE Trans. Compon. Packag. Technol..

[16] Tian W., Zhang S., Li W., Chen Y., Zhao J., Xin F., Qian Y., Li W. (2023). Study on Cavitation, Warpage Deformation, and Moisture Diffusion of Sop-8 Devices during Molding Process. Micromachines.

[17] Xu H., Huang J., Tian W., Li Z. (2023). Thermal Performance Optimization of Integrated Microchannel Cooling Plate for IGBT Power Module. Micromachines.

[18] Yu D.-J., Chen X., Chen G., Lu G.-Q., Wang Z.-Q. (2009). Applying Anand model to low-temperature sintered nanoscale silver paste chip attachment. Mater. Des..

[19] Brown S.B., Kim K.H., Anand L. (1989). An internal variable constitutive model for hot working of metals. Int. J. Plast..

[20] Chen G., Zhang Z.-S., Mei Y.-H., Li X., Yu D.-J., Wang L., Chen X. (2014). Applying viscoplastic constitutive models to predict ratcheting behavior of sintered nanosilver lap-shear joint. Mech. Mater..

[21] Gao S., Wang R., Wang H., Kang R. (2023). Warping model of high-power IGBT modules subjected to reflow soldering process. Int. J. Mech. Sci..

[22] Sun W., Wang L., Zhu N., Xin J., Luo Y., Jiang X., Fan G., Chen M. (2023). Characterization of packaging warpage, residual stress and their effects on the mechanical reliability of IGBT power modules. Eng. Fail. Anal..

[23] Fuste N., Avino O., Perpina X., Sanchez D., Vellvehi M., Jorda X. Determination of Anand viscoplastic constitutive parameters for the AuGe solder alloy from experimental stress-strain curves for power systems integration FEA simulations. Proceedings of the 2021 Smart Systems Integration (SSI).

[24] Haouala S., Doghri I. (2015). Modeling and algorithms for two-scale time homogenization of viscoelastic-viscoplastic solids under large numbers of cycles. Int. J. Plast..

[25] Holopainen S., Barriere T., Cheng G., Kouhia R. (2017). Continuum approach for modeling fatigue in amorphous glassy polymers. Applications to the investigation of damage-ratcheting interaction in polycarbonate. Int. J. Plast..

[26] Guo Y., Liu M., Yin M., Yan Y. (2022). Reliability sensibility analysis of the PCB assembly concerning warpage during the reflow soldering process. Mathematics.

[27] Li J., Johnson C.M., Buttay C., Sabbah W., Azzopardi S. (2015). Bonding strength of multiple SiC die attachment prepared by sintering of Ag nanoparticles. J. Mater. Process. Technol..

[28] Qi K., Chen X., Lu G.Q. (2008). Effect of interconnection area on shear strength of sintered joint with nano-silver paste. Solder. Surf. Mt. Technol..

[29] Lu X., Lv Z., Sun Y., Murugesan M., Zhou C., Zhang X., Liu J. (2022). Enhanced Mechanical and Thermal Properties of Ag Joints Sintered by Spark Plasma Sintering. J. Electron. Mater..

